# Elevated Plasma Protein Carbonyl Concentration Is Associated with More Abnormal White Matter in People with HIV

**DOI:** 10.3390/v15122410

**Published:** 2023-12-12

**Authors:** Patricia K. Riggs, Albert M. Anderson, Bin Tang, Leah H. Rubin, Susan Morgello, Christina M. Marra, Benjamin B. Gelman, David B. Clifford, Donald Franklin, Robert K. Heaton, Ronald J. Ellis, Christine Fennema-Notestine, Scott L. Letendre

**Affiliations:** 1 Division of Infectious Diseases and Global Public Health, Department of Medicine, University of California San Diego, San Diego, CA 92093, USA; 2 Division of Infectious Diseases, Department of Medicine, Emory University School of Medicine, Atlanta, GA 30322, USA; 3 Department of Psychiatry, University of California San Diego, San Diego, CA 92093, USA; 4 Departments of Neurology, Psychiatry and Behavioral Sciences, and Epidemiology, The Johns Hopkins University, Baltimore, MD 21205, USA; 5 Departments of Neurology, Neuroscience, and Pathology, Mt Sinai School of Medicine, New York, NY 10029, USA; 6 Department of Neurology, University of Washington, Seattle, WA 98195, USA; 7 Departments of Pathology, and Neuroscience & Cell Biology, University of Texas Medical Branch, Galveston, TX 77555, USA; 8 Department of Neurology, Washington University in St Louis, St Louis, MO 63110, USA; 9 Department of Neurosciences, University of California San Diego, San Diego, CA 92093, USA; 10 Department of Radiology, University of California San Diego, San Diego, CA 92093, USA

**Keywords:** HIV, oxidative stress, brain, magnetic resonance imaging, white matter

## Abstract

Structural brain abnormalities, including those in white matter (WM), remain common in people with HIV (PWH). Their pathogenesis is uncertain and may reflect multiple etiologies. Oxidative stress is associated with inflammation, HIV, and its comorbidities. The post-translational carbonylation of proteins results from oxidative stress, and circulating protein carbonyls may reflect this. In this cross-sectional analysis, we evaluated the associations between protein carbonyls and a panel of soluble biomarkers of neuronal injury and inflammation in plasma (N = 45) and cerebrospinal fluid (CSF, *n* = 32) with structural brain MRI. The volume of abnormal WM was normalized for the total WM volume (nAWM). In this multisite project, all regression models were adjusted for the scanner. The candidate covariates included demographics, HIV disease characteristics, and comorbidities. Participants were PWH on virally suppressive antiretroviral therapy (ART) and were mostly white (64.4%) men (88.9%), with a mean age of 56.8 years. In unadjusted analyses, more nAWM was associated with higher plasma protein carbonyls (*p* = 0.002) and higher CCL2 (*p* = 0.045). In the adjusted regression models for nAWM, the association with plasma protein carbonyls remained significant (FDR *p* = 0.018). Protein carbonyls in plasma may be a valuable biomarker of oxidative stress and its associated adverse health effects, including within the central nervous system. If confirmed, these findings would support the hypothesis that reducing oxidative stress could treat or prevent WM injury in PWH.

## 1. Introduction

Neuropsychiatric complications remain common among people with HIV (PWH) who are taking effective antiretroviral therapy (ART) [1,2]. Neuroimaging provides a window into brain health in PWH. With virologic control during ART, the changes seen on macrostructural magnetic resonance imaging (MRI) have become more subtle and localized, especially gray matter volume loss [3]. Increased white matter (WM) abnormalities (e.g., hyperintense regions on T2-weighted images) persist in PWH relative to sociodemographically similar people without HIV, and these progress despite ART [4,5,6,7]. WM abnormalities are associated with aging, cognitive impairment, depression, and worse daily functioning in the general population [8,9,10,11,12,13] and in PWH [14,15,16,17,18,19]. As PWH age, more research is needed to understand the risk factors and biological mechanisms of WM abnormalities. Such information should support efforts to prevent and treat neuropsychiatric complications in PWH. Further, diagnostic or prognostic biomarkers in blood would be particularly helpful in the clinic.

In the general population, WM abnormalities are typically attributed to cerebral small vessel disease (CSVD). While PWH have a higher burden of cerebrovascular disease than people without HIV [20], this does not fully explain the increase in WM abnormalities [6,17,20,21]. Additionally, debate continues with respect to whether HIV itself or HIV-associated comorbid conditions are the principal cause [4,18,22,23,24,25]. Prior studies have linked WM disease to both HIV disease-related factors (e.g., lower CD4+ T-cell nadir, longer duration of HIV infection, as well as CD4+ T-cell recovery) and comorbidities (e.g., hepatitis C virus (HCV) seropositivity, diabetes, and cerebrovascular risk factors) [4,23,24,26,27]. In sum, current data support a multifactorial etiology of WM abnormalities in PWH, even in the setting of effective ART.

Oxidative stress is one mechanism by which the brain can be damaged in the setting of HIV—and a pathway shared by many of the other risk factors for WM disease (e.g., aging, diabetes, and cardiovascular disease). PWH, including those on virally suppressive ART, exhibit elevated oxidative stress which has been implicated in neuropsychiatric complications [28,29,30,31,32,33]. Oxidative stress occurs when the production of oxidants exceeds antioxidant capacity. Therefore, increased oxidation, decreased antioxidant quantity or function, or impaired repair and removal of end products of oxidation can lead to oxidative stress. HIV may affect each of these factors [31,33,34,35,36,37], although the strongest evidence is for increased oxidant production [38]. HIV proteins, including gp120 and Tat, can induce the intracellular production of reactive oxygen species, including in microglia, neurons, and astrocytes [31,39,40]. The production of reactive oxygen species is especially pronounced with the depletion of CD4+ T-cells [36]. Oxidative stress biomarkers are also elevated in post-mortem brain tissue from PWH [33,41]. More recently, transcriptomic analysis of monocytes and metabolomics analysis of serum demonstrated that increased oxidative stress may be a key driver of increased inflammasome activation, specifically in PWH on suppressive ART [37]. Additionally, ART itself may cause oxidative stress [31,34,35,42].

Oxidative stress can be measured in several ways, including by end products of oxidation (e.g., oxidized proteins, lipids, or nucleic acids). Protein carbonyls are created by post-translational modification via oxidation of amino acid residues, and they are one biomarker of oxidative stress. Proteins can be carbonylated by reactive oxygen species directly or by-products of lipid oxidation. This irreversible modification can alter protein function and requires degradation to avoid accumulation and further toxicity and inflammation [43,44,45]. Few studies report data on protein carbonyl concentrations in PWH. One of these found increased protein carbonyls in CSF in PWH with dementia [46]. In the general population, higher plasma protein carbonyls are associated with Alzheimer’s disease [47,48] and schizophrenia [49] as well as non-neurologic conditions characterized by chronic inflammation, including rheumatoid arthritis, diabetes mellitus, and vascular disease [28,44,50]. While oxidative stress is not specific to HIV, assessing it can expand understanding of biological mechanisms underlying comorbid conditions in PWH and may have implications for therapeutics [51,52,53,54].

In the present study, we examined the relationship of soluble biomarkers of oxidative stress, neurodegeneration, and immune activation in plasma and CSF with global measures of brain structure using MRI in PWH. We hypothesized that the concentrations of these biomarkers would be more abnormal in individuals with more abnormal WM.

## 2. Materials and Methods

Participants. We analyzed cross-sectional data from 45 PWH who had a structural brain MRI and a panel of biomarkers measured in plasma, in addition to a standardized battery of neuromedical and laboratory assessments in the CHARTER (CNS HIV ART Effects Research) Aging project between 2016 and 2019 [55]. All participants were taking ART and had plasma HIV RNA below 200 copies/mL. A subgroup of 32 participants also underwent lumbar puncture and had biomarkers measured in CSF. CHARTER is a prospective, observational study and five CHARTER sites contributed MRI data (Baltimore, *n* = 8; Galveston, *n* = 11; New York, *n* = 9; San Diego, *n* = 13; and Seattle, *n* = 4). In the CHARTER cohort, prospective participants were excluded for active opportunistic infections, severe psychiatric disorders (e.g., untreated schizophrenia), drug use that would interfere with participation, or for inability to complete the assessments [1]. All procedures were approved by an Institutional Review Board. All participants provided written informed consent.

Laboratory Assessments. All participants had a diagnosis of HIV at enrollment that was confirmed with an enzyme-linked immunosorbent assay. HIV RNA in plasma was quantified using a commercial assay with a lower limit of quantification (LLOQ) of 20 copies/mL; clinical viral suppression was defined as less than 200 copies/mL as per United States Department of Health and Human Services guidelines [56]. Peripheral blood T cell subsets were measured using flow cytometry performed by a Clinical Laboratory Improvement Amendments (CLIA)-certified laboratory.

Soluble biomarkers were measured using either a commercial immunoassay or bead suspension array (Millipore, Burlington, MA, USA for CD40L only). Protein carbonyls (Cell Biolabs, San Diego, CA, USA, LLOQ 0.375 nmol/ng), 8-hydroxydeoxyguanosine [8-OHdG] (Trevigen, Gaithersburg, Maryland, USA; LLOQ 3.13 nM), neopterin (ALPCO, Salem, NH, USA; LLOQ 6.4 nmol/L), amyloid β 1–42[Aβ 1–42] (MSD, Rahway, NJ, USA; LLOQ 0.168 pg/mL), soluble amyloid precursor protein-α [sAPPα] (MSD, LLOQ 0.01 ng/mL), interleukin-6 [IL-6] (MSD, LLOQ 0.17 pg/mL), CCL2 (MSD; LLOQ 0.129 pg/mL), soluble tumor necrosis factor receptor II [sTNFR-II] (MSD; LLOQ 0.61pg/mL), and soluble CD14 [sCD14] (R&D Systems, Minneapolis, MN, USA; LLOQ 250 pg/mL) were measured in both plasma and CSF; C-reactive protein [CRP] (MSD; LLOQ 12.5 pg/mL), D-dimer (BioMedica, Vienna, Austria; LLOQ 12 ng/mL), and soluble CD40 ligand [sCD40L] (bead suspension array, Millipore; LLOQ 3.2 pg/mL) were measured in plasma only; neurofilament light chain [NfL] (TECAN, Männedorf, Switzerland; LLOQ 100 pg/mL) and total Tau (MSD; LLOQ 14.7 pg/mL), were measured in CSF only. All results were reviewed for quality assurance and assays were repeated when coefficients of variation exceeded 20% or if concentration distributions revealed possible batch effects. This list of biomarkers represents a select list of biomarkers of inflammation, oxidative stress, cardiovascular disease, and neurodegeneration. Plasma and CSF 8-OhdG, protein carbonyls, plasma CRP, d-dimer, sCD40L, sAPP, and Amyloid β 1–42 were analyzed given their associations with aging, inflammation, dementia, AD, and neurocognitive impairment [28,57,58,59,60,61,62,63]. sCD14, neopterin and sTNFRII were analyzed for their role in microglial signaling and response to neuronal injury [64,65]. Amyloid β 1–42 and NfL were analyzed in CSF due to previous findings by our group and others demonstrating an association between CSF oxidative damage and these two biomarkers in the CSF, but not in the plasma [38,66].

Magnetic Resonance Imaging. T1-, T2-, and proton density-weighted volumes were acquired at each site using a standardized protocol, all image processing was conducted at one location, and differences associated with scanner vendor hardware were addressed within statistical analyses as in prior work (see below and references [12,26,67]). All data were processed with a multichannel segmentation approach for volumetric measurement of abnormal WM (AWM), total WM, GM, CSF, and intracranial vault volume (ICV), as previously described [12]. This approach leverages complementary information in three volumes to increase measurement sensitivity while reducing the impact of acquisition noise. The primary AWM measure includes areas within the white matter that are hypointense on T1 and hyperintense on T2 and PD images; this is comparable to the measure commonly referred to as ‘white matter hyperintensities (WMH)’ that refer to the signal value on the T2. Steps include standard alignment of the T1-weighted image (i.e., 6 degrees-of-freedom and rigid transformation to an anterior/posterior commissure-aligned space), co-registration of the T1-, T2-, and proton density-weighted volumes using a mutual information method [68], intensity non-uniformity correction using N3 [69], and a three-class tissue segmentation using Scott’s L2E method [70] to determine robust means and covariances for WM, GM, and CSF. AWM was classified using morphological operators [71] to identify voxel clusters originally segmented as GM that fell within anatomically defined WM regions. Results were visually reviewed and manually edited when necessary to correct misclassifications. Since partial voluming of CSF and WM along the edges of ventricles results in voxels with AWM-like signals, even in healthy individuals, we did not allow any voxels that touched (i.e., shared a common face, edge, or vertex) a ventricular fluid voxel to be classified as AWM; such voxels were excluded from the estimated volumes.

Statistical Analyses. Demographics, HIV disease characteristics, and comorbidities were summarized with mean and standard deviation or median and interquartile range for continuous variables, and numbers and percentages for categorical variables. Volumetric imaging data were normalized for analysis to account for individual differences in head size. Specifically, normalized AWM (nAWM) is the total abnormal WM volume normalized for total WM volume and normalized GM (nGM) is the total GM volume normalized for intracranial volume. To account for differences driven by scanner-related factors (e.g., vendor-related hardware), all regression analyses include the scanner [26,67]. MRI outcome variables (e.g., nAWM) and biomarker measurements were transformed to reduce skewness and stabilize distributions.

Relationships between soluble biomarkers and MRI outcome measures were first assessed with Pearson’s correlation analysis. Next, associations between nAWM and individual variables of interest were evaluated using regression, adjusting for the scanner only. The variables were selected based on a literature review and prior work by our group to include factors previously associated with AWM, protein carbonyls, or oxidative stress. These variables included demographic characteristics, HIV disease characteristics (CD4+ T-cell count, CD4+ T-cell nadir, and estimated duration of HIV infection) [4,23,24,26,27], HCV serostatus [4,26], diabetes [25], hypertension [4,14,18], body mass index (BMI) [72], hyperlipidemia [73], Framingham cardiovascular risk score [74], and current and lifetime tobacco use [4,75,76]. Variables predictive of nAWM with *p* < 0.2 on regression, adjusting for the scanner, were then evaluated using multivariable regression modeling. The final, reduced model that predicted nAWM was determined using backward stepwise elimination using Akaike Information Criterion (AIC) as the selection criterion. In addition, we identified associations between plasma protein carbonyls and other biomarkers in plasma and CSF using simple linear regression. A significance level α was set to 0.05. Analyses were performed using R software (version 4.2.1) and JMP Pro (version 16.0.0). The false discovery rate (FDR) method was used to reduce type I errors in multivariable models.

## 3. Results

Participant characteristics. Table 1 summarizes the characteristics of the 45 participants, who were majority white (64.4%) men (88.9%) with a median age of 55 years and had HIV for a median of 24.2 years. The median nadir CD4+ T-cell count was under 200 cells/µL and the median count at the time of the assessment was over 500 cells/µL. More than half of the participants were HCV seropositive. Most participants (78.8%) had smoked tobacco in their life, but only seven (15.6%) reported tobacco use at the time of the assessment.

**Figure 1:**
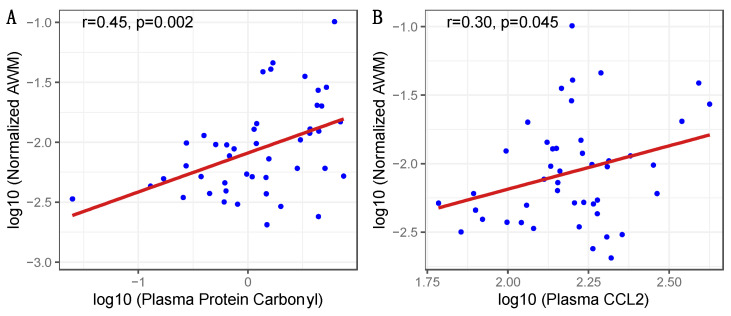
Scatter plots for significant correlations between normalized abnormal white matter and soluble biomarkers. Log transformed values of normalized AWM (abnormal white matter volume/total white matter volume) by (**A**) plasma protein carbonyl concentration and (**B**) plasma CCL2 levels. Shaded bands represent 95% confidence intervals.

Relationships between biomarkers and imaging. Pearson’s correlation analysis identified that higher nAWM was correlated with higher plasma protein carbonyls (r = 0.45, *p* = 0.002) and plasma CCL2 (r = 0.30, *p* = 0.045), as shown in Figure 1. There were non-significant trends between nGM and higher plasma sCD40L (r = −0.29, *p* = 0.051) and lower CSF NFL (r = −0.32, *p* = 0.079).

We then further evaluated predictors of nAWM with limited regression models that included a single variable of interest and a scanner. When adjusting for the scanner, the association between nAWM and plasma protein carbonyls remained significant (std β = 0.423, *p* = 0.0016). The weaker correlation between nAWM and plasma CCL2 was no longer significant in regression analysis when accounting for the scanner (std β = 0.174, *p* = 0.274). Individual variables predictive of nAWM with a *p*-value < 0.2 included tobacco use, race, HCV serostatus, plasma D-Dimer, plasma CRP, and age, as shown in Table 2. These variables were included in the backward stepwise regression for model selection and only plasma protein carbonyls, age, lifetime tobacco use, and plasma CRP remained in the model as significant predictors of nAWM. This model had an R^2^ = 0.51 and *p* < 0.0001.

Other variables of interest which were not associated with nAWM included the other soluble plasma biomarkers, HIV disease characteristics (current CD4, CD4 nadir, history of AIDS diagnosis, estimated duration of HIV, and duration of current ART regimen) and other clinical characteristics and comorbid conditions (HCV, diabetes, hemoglobin A1c, hypertension, hyperlipidemia, BMI, and Framingham cardiovascular risk score). Female sex was originally identified as a candidate for multivariable analysis for predicting nAWM with a *p* = 0.104 in regression adjusting for the scanner. However, there were only five women at only two of the five sites included in this cohort. Univariable regression without correcting for the scanner did not find any association between nAWM and sex. CSF-soluble biomarkers were available for a subset (*n* = 32) and none of these were associated with nAWM.

**Figure 2:**
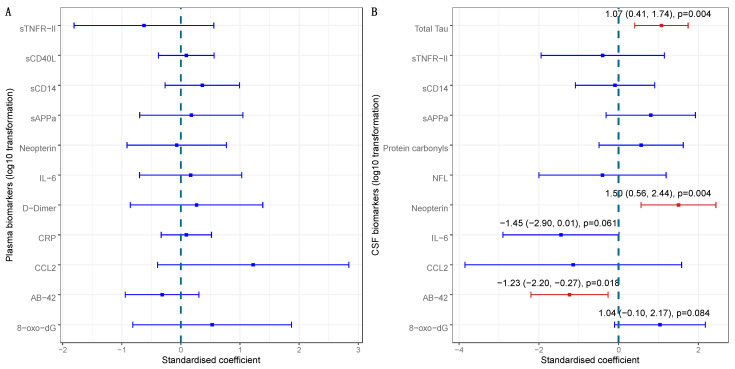
The effect of biomarkers in plasma and CSF on plasma protein carbonyl concentrations. Univariable analyses were carried out with regressing plasma protein carbonyl on plasma biomarkers with N = 45 (**A**) and CSF biomarkers with N = 32 (**B**).

Relationships between plasma protein carbonyls and other biomarkers. Based on these observations, we assessed the relationships between plasma protein carbonyls and the other biomarkers (Figure 2). The plasma concentration of protein carbonyls was not associated with concentrations of other plasma biomarkers (Figure 2A), but a higher plasma concentration of protein carbonyls was associated with higher CSF concentrations of total Tau [standardized β = 1.07, *p* = 0.004] and CSF neopterin [standardized β = 1.50, *p* = 0.004], and a lower CSF concentration of Aβ-42 [standardized β= −1.23, *p* = 0.018] (Figure 2B).

## 4. Discussion

The principal finding of these analyses of the soluble biomarkers and structural brain MRIs of 45 PWH on virally suppressive ART is that greater circulating protein oxidation was associated with more abnormal WM. Protein carbonylation is one of several nonenzymatic post-translational modifications that are caused by oxidation, and increases in their concentration in plasma are seen in aging and aging-related diseases, including vascular disease, diabetes mellitus, and Alzheimer’s disease [43,47,77,78,79]. Similarly, WM abnormalities are associated with aging and aging-related diseases, which supports the validity of the current findings [5,9,12,13] although the cross-sectional design does not allow causal inference. An increase in plasma protein carbonyl concentration has been associated with abnormal white matter in people with schizophrenia [49] and elevated tissue concentrations of protein carbonyls have been found in the white matter plaques of individuals with multiple sclerosis [80]. The findings from the current study therefore provide validation of a link between protein carbonylation and white matter disease, but this is the first study to our knowledge in linking oxidative stress, specifically plasma protein carbonyls, to white matter abnormalities in PWH.

Proteins can be carbonylated by either reactive oxygen species directly or by-products of lipid oxidation. This process results in an irreversible modification that can alter protein function and requires degradation by proteolysis to avoid accumulation and further toxicity and inflammation [43,44,45]. Furthermore, the enzymes required for proteolysis may be carbonylated and inactivated and, in the setting of sustained oxidative stress, this may lead to further accumulation of abnormal proteins and promotion of inflammation [81,82]. Technically, an increased level of protein carbonylation indicates consequences of oxidative stress but does not distinguish between increased production of reactive oxygen species, reduced antioxidant capacity, or impaired proteasomal function. However, a combination of all three likely contributes [31,33,34,35,36,37]. Additionally, in this cross-sectional study of all PWH on suppressive ART, we cannot determine the cause(s) of oxidative stress or the degree to which HIV contributes.

The associations between plasma protein carbonyls with CSF neopterin, Aβ 1–42, and total Tau in the subgroup of 32 participants with CSF biomarkers further suggest a role for protein carbonylation in neuroinflammation and injury, but they could also simply be a convergent indicator of underlying neuropathology. Neopterin is a nonspecific biomarker of inflammation that is produced in many cell types, especially myeloid cells, in response to interferon-γ. CSF neopterin has previously been associated with white matter abnormalities [65] and cognitive impairment in PWH [83,84], as well as with both oxidative stress and neurodegeneration [85], further supporting the validity of our findings. The associations with biomarkers of neurodegeneration (higher CSF total Tau, lower CSF Aβ 1–42) are consistent with prior studies linking oxidative stress and Alzheimer’s disease-type pathology in PWH [38,47,64,81,86,87,88,89,90]. However, from this study, we cannot determine if this is related to white matter abnormalities or a reflection of the nonspecific nature of oxidative stress.

Another end product of oxidative stress, 8-OHdG, is a marker of DNA oxidation, and CSF 8-OHdG has previously been associated with a higher concentration of buccal mitochondrial DNA, higher CSF NFL, and lower CSF Aβ 1–42 in PWH [38,91]. In the present analysis, neither plasma nor CSF 8-OHdG was associated with protein carbonyls or nAWM. This may be because, unlike protein carbonylation, DNA oxidation is a dynamic, reversible modification; it can be reversed by antioxidants [46,92,93] and repaired by DNA repair mechanisms [93]. Thus, 8-OHdG may better reflect acute oxidative stress while protein carbonyls may indicate chronic oxidative stress [44,93,94].

Consistent with prior findings, older age, history of tobacco use, and higher plasma CRP were also associated with nAWM and were selected in the final multivariable model for nAWM [4,19]. Plasma protein carbonyl levels remained the strongest predictor of nAWM in univariable and multivariable analyses. Surprisingly, nAWM was not associated with other comorbidities and more modifiable risk factors typically associated with small vessel cerebrovascular disease (hypertension, hyperlipidemia, diabetes mellitus, hemoglobin A1c, BMI, or Framingham cardiovascular disease risk score). In the context of limited power due to the small sample size, these factors still likely are associated with AWM as shown previously [4,12,25,73]. It is possible that with multifactorial etiologies of WM abnormalities, a nonspecific marker like plasma protein carbonyl levels is an aggregate measure of several different etiologic factors.

Unlike prior reports from the CHARTER cohort, our analyses did not find associations between nAWM and the duration of HIV infection, current or nadir CD4+ T-cell count, or HCV serostatus. One reason may be limited power (N = 45) and the need to account for the influence of different scanners in analyses. However, there is also increasing evidence for attenuation of the effect of pretreatment immunocompromise—and the increased role of comorbid conditions—on neuropsychiatric complications for PWH on ART so this needs to be evaluated in a larger study [55,95]. HCV seropositivity had a trend level association with nAWM when adjusting for the scanner alonebut was not significant in multivariable analyses. As in prior studies, our analyses were based on HCV serostatus, without distinguishing between chronic and resolved HCV infection. Future studies would benefit from HCV RNA measurement which would improve power to detect an HCV effect.

The association with plasma protein carbonyl levels, and lack of association with HIV disease characteristics, provide additional evidence that WM abnormalities reflect an active process that continues on virally suppressive ART rather than a legacy effect from a remote, discreet insult during prior severe immunosuppression, opportunistic infections, or uncontrolled viremia. This distinction emphasizes the potential for therapeutic intervention in an active pathological process.

It is also important to note a few key differences between this subgroup and the larger CHARTER aging cohort [55]. Overall, this subset is similar in terms of demographics, depression history, substance use history, and rate of neurocognitive impairment (defined as a global deficit score > 0.5) [96,97]. However, this subgroup has a higher percentage of individuals with a history of severe immunocompromise (89% with a prior AIDS diagnosis), and half (53%) were HCV seropositive. Thus, our results may not be generalizable to the entire PWH population and need further study.

Readers should also consider other limitations of this study. It is important to reiterate that this is a cross-sectional analysis and therefore causality cannot be inferred. We did not find associations between imaging measures and CSF biomarkers, but these subgroup analyses were hampered even more by the smaller sample size (*n* = 32). While we recognize the importance of sex as a biological variable in research, analyses of sex were not feasible in this study with only five women at only two of the five sites included in this cohort with all measures available. This may be particularly relevant, for example, because plasma protein carbonyls are only associated with Alzheimer’s disease in men [48,98]. Due to the small sample size and heterogeneity of ART regimens, we were not able to examine differences in biomarkers or imaging measures by the type of ART, which is an important gap in the current knowledge [31,34,35]. However, the limitations in power and accounting for Type 1 errors with false discovery rates in the final multivariable model underscores the strength of the association between plasma protein carbonyls and nAWM.

Larger studies with both PWH and people without HIV are needed to confirm our findings, to determine the degree to which HIV modifies the observed relationships, and to better understand the role of the type of ART, comorbidities, sex, social determinants of health, exercise, and antioxidant intake. Ongoing efforts for improved standardization and data harmonization for MR research protocols and the inclusion of more functional and microstructural assessments will allow for larger studies.

Our findings provide support for measuring protein carbonyl concentration in plasma in imaging studies, especially in PWH and white matter disease. In addition, broadening the panel of oxidative stress-related biomarkers and linked ex vivo assessments should also provide more insight into the precise mechanisms linking end products of oxidative stress with immune activation and neurotoxicity [47] and help identify potential therapeutic targets. For example, future directions could include assessments of (1) the mechanisms leading to the production of reactive oxygen species; (2) measurement of antioxidant capacity and antioxidant intake; (3) other oxidation-related post-translational modifications (e.g., glycation); (4) other oxidized molecules (e.g., lipids perioxidation, malondialdehyde, and 4-Hydroxy-2-nonenal); (5) degradation of modified proteins (e.g., proteasome 20S function); and (6) comprehensive proteomic analysis to determine if specific proteins are more prone to carbonylation in PWH. Next steps also include evaluating associations between clinical neuropsychiatric outcomes (e.g., cognition and depression), AWM, and oxidative stress in larger groups in CHARTER and other cohorts.

In addition to biological and mechanistic implications, our findings have clinical and therapeutic implications. The observed associations with protein carbonyls in an easily accessible body fluid (blood) make the findings more clinically accessible than a biomarker in CSF or imaging. It is important to recognize that plasma protein carbonyls are not a specific marker unique to AWM in PWH. However, nonspecific tests provide valuable, clinically actionable information every day. This study is consistent with the existing literature that supports the potential diagnostic value of protein carbonyls measured in blood [28,43,44,47,48,49,50]. Further, protein carbonyls are easily quantified with a simple immunoassay. While the assay is not approved for clinical use, it is well-suited for high throughput testing like other clinical immunoassays. A better understanding of oxidation-related biological mechanisms in PWH could identify new therapeutic targets.

## 5. Conclusions

In conclusion, protein carbonyls may be a valuable blood biomarker of oxidative stress and its associated adverse health effects, including neuropathology. Including the measurement of plasma protein carbonyls in future and ongoing neuroimaging studies may provide important insights into the role of protein oxidation in persistent neuroinflammation and neurotoxicity, particularly in aging PWH [45,54,79,81,82,99]. Reducing oxidative stress, regardless of the cause, could potentially mitigate additional damage.

## Figures and Tables

**Table 1:** Demographic, HIV disease, and comorbidity characteristics. Values reported are either number (%) or median [IQR]. N = 45. All participants were taking ART and had plasma HIV RNA ≤ 200 copies/mL.

Demographic Characteristics	
Age (years)	55 [52, 63]
Sex (male)	40 (88.9%)
Race	
White	29 (64.4%)
Black	16 (35.6%)
Ethnicity (Hispanic)	6 (13.3%)
Education (years)	13 [12, 15.5]
**HIV Disease Characteristics**	
CD4+ T Cell Count (cells/μL)	527 [387, 810]
CD4+ T Cell Nadir (cells/μL)	104 [19.5, 190]
AIDS Diagnosis	40 (88.9%)
Estimated Duration of HIV (years)	24.2 [17.0, 29.5]
Duration of Current Regimen (months)	25.3 [8.2, 72.0]
Duration of All ART (months)	188 [150, 239]
ART Regimen Class	
Integrase Inhibitor Use	27 (60.0%)
Protease Inhibitor Use	13 (28.9%)
Non-nucleoside Reverse Transcriptase Inhibitor Use	11 (24.4%)
**Comorbid Conditions**	
Body Mass Index (kg/m^2^)	25.7 [23.3, 29.8]
HCV Seropositive	24 (53.3%)
Diabetes Mellitus	8 (17.8%)
Hypertension	25 (55.6%)
Hyperlipidemia	19 (42.2%)
Framingham 10-year Risk Score	15.7% [8.9, 24.0]
Chronic Pulmonary Disease	8 (17.8%)
Current Substance Use Disorder	2 (4.6%)
Lifetime Substance Use Disorder	37 (82.2%)
Current Tobacco Use	7 (15.6%)
Lifetime Tobacco Use	35 (77.8%)
Current Major Depression	2 (4.6%)
Lifetime Major Depression	31 (68.9%)
Neurocognitive Impairment (global deficit score ≥ 0.5)	17 (37.8%)

**Table 2:** Regression table of normalized abnormal white matter. Scanner only refers to nAWM regressed on the scanner and listed variables. Variables with *p* < 0.2 were then included in multivariable linear regression modeling. Multivariable model selection was performed using the Akaike Information Criterion (AIC) and backward elimination. Model R^2^ for the best fit is 0.51, model *p* < 0.0001.

	Scanner Only		Multivariable			
	Std β	*p* Value	Std β	*p* Value	FDR *p* Value	Risk Direction
Plasma Protein Carbonyls ^a^	0.423	0.002	0.340	0.006	0.018	Higher
Lifetime Tobacco Use	0.378	0.016	0.276	0.028	0.042	Present
Race	0.404	0.035				Black
HCV Serostatus	0.286	0.074				Positive
Plasma D-Dimer ^a^	0.252	0.097				Higher
Current Tobacco Smoking	0.226	0.116				Present
Plasma C-Reactive Protein ^a^	0.225	0.123	0.239	0.049	0.059	Higher
Age	0.213	0.176	0.312	0.023	0.042	Older

CI—confidence interval; Std β—Standardized βeta; FDR—False Discovery Rate. ^a^ log10 transformed.

## Data Availability

The data that support the findings of this study are available to the public on request from the National NeuroAIDS Tissue Consortium (NNTC) Data Coordinating Center (https://nntc.org/content/requests), which coordinates requests for data and biospecimens from the CHARTER resources. The data are not available without a formal request since the NNTC/CHARTER Steering Committee reviews requests for scientific merit and tracks and reports use of the resources to funding agencies.
